# Susceptibility phantom for compatibility testing of SPECT components for a SPECT/MR hybrid system

**DOI:** 10.1186/2197-7364-1-S1-A25

**Published:** 2014-07-29

**Authors:** Jan Rieger, Roman Leicht, Helmar Waiczies, Darius Lysiak, Celal Oezerdem, Magor Babos, Thoralf Niendorf

**Affiliations:** MRI.TOOLS GmbH, Berlin, Germany; Berlin Ultrahigh Field Facility (B.U.F.F.), Max-Delbrueck-Center for Molecular Medicine, Berlin, Germany; Mediso Medical Imaging Systems, Budapest, Hungary; Experimental and Clinical Research Center (ECRC), a joint cooperation between the Charité Medical Faculty and the Max-Delbrueck Center, Berlin, Germany

**Figure 1:**
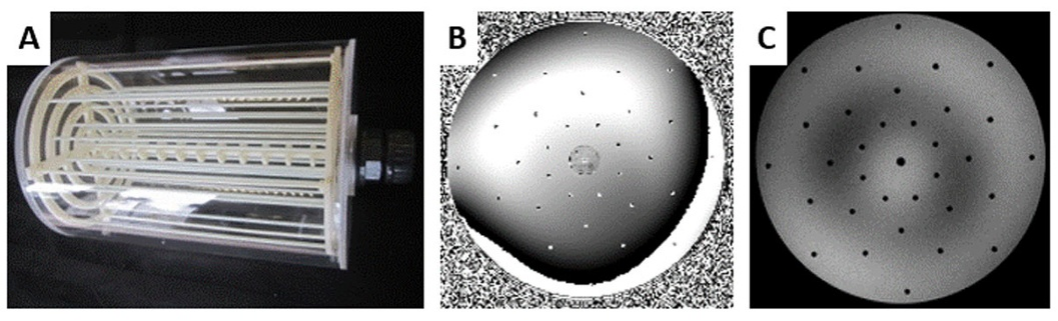
A) Susceptibility imaging phantom without the agarose gel B) Reference phase image of the phantom at d = 30 mm from the front end of the phantom C) Reference magnitude image of the phantom at d= 30 mm from the front end of the phantom

A susceptibility phantom was designed and built from an acrylic glass cylinder (inner diameter: 170 mm, length: 250 mm). A reference structure consisting of 32 rods (diameter: 3 mm) was placed inside the phantom (Figure 1A) together with a plastic scale with marks every 20 mm. The phantom was filled with agarose gel (ε_r_ = 75, σ = 0.73 S/m) to mimic human tissue [2].

**Figure 2:**
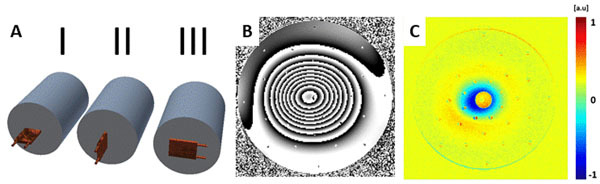
A) Illustration of the orientation of the copper cooling block in respect to the phantom B) Susceptibility effects in the phase image caused by the cooling block in orientation III, d=30 mm C) Distortion in the magnitude image caused by the cooling block in orientation III, d=30 mm

Amplitude and phase images were acquired with a gradient echo technique at 7.0 T (5 echoes TE=5/10/15/20/25/30 ms). A reference image was acquired with the phantom placed longitudinally in the magnets isocenter without the object under test (OUT). Subsequently, the OUT was positioned at the front end of the phantom (Figure 2A) and a series of tranversal images covering the entire phantom was acquired. A post-processing algorithm was used to evaluate image. This setup was employed to examine the MR compatibility of a copper cooling block (52 x 74 x 8 mm³).

No distortion was found to be at d = 30 mm, d = 30 mm and d = 200 mm for three different orientations of a copper cooling block.

The proposed setup supports the assessment of susceptibility effects induced by SPECT components. Our results demonstrate that the orientation and position of SPECT components in an MR environment need to be carefully taken into account during the design process of an integrated SPECT/MR device to avoid image distortion.
